# Approach to urinary tract infections

**DOI:** 10.4103/0971-4065.65311

**Published:** 2010-04

**Authors:** S. Aggarwal, H. Singh

**Affiliations:** University College of Medical Sciences, New Delhi, India; 1 All India Institute of Medical Sciences, New Delhi, India

Sir,

We read the article “Approach to urinary tract infections”[1] by Najar *et al*. with interest. We appreciate the extensive research conducted by the authors to come up with this review article. We would like to add our views to the same.

The authors fail to mention the importance of treatment for both gonorrhea and Chlamydia with a third-generation cephalosporin and macrolide, respectively, simultaneously in a patient with urethritis. Failing to do so may result in a high chance of recurrence of the symptoms and dreadful complications like stricture and infertility at a later stage. Thus, care should be taken to treat for both in the concerned patients. Nonetheless, the authors have performed a commendable job and need to be applauded for the same.
